# Lesser-Explored Edible Flowers as a Choice of Phytochemical Sources for Food Applications

**DOI:** 10.1155/2024/9265929

**Published:** 2024-11-12

**Authors:** Mariel Guadalupe Valencia-Cordova, Yari Jaguey-Hernández, Araceli Castañeda-Ovando, Luis Guillermo González-Olivares, E. Pedro Castañeda-Ovando, Javier Añorve-Morga, Minarda de la O-Arciniega

**Affiliations:** ^1^Chemistry Department, Autonomous University of Hidalgo State, Mineral de la Reforma, Hidalgo 42184, Mexico; ^2^Agroindustry Engineering Department, Polytechnque University of Francisco I. Madero, Francisco I. Madero, Hidalgo 42660, Mexico; ^3^Autonomous University of Hidalgo State, Institute of Health Sciences, San Agustín Tlaxiaca, Hidalgo 42160, Mexico; ^4^Autonomous University of Hidalgo State, Institute of Basic Sciences and Engineering, Mineral de la Reforma, Hidalgo 42184, Mexico

**Keywords:** bioactive compounds, edible flowers, floriphagia, health benefits, lesser-explored

## Abstract

Flowers have been commonly used in cooking to add color and flavor to dishes. In addition to enhancing the visual appeal of food, many edible flowers also contain bioactive compounds that promote good health. These compounds include antimicrobial, antihypertensive, nephroprotective, antiulcer, and anticancer agents. In the last 5 years, there have been 95 published reviews about edible flowers. Among these, 43% have concentrated on Food Science and Technology, while 32% have analyzed their effects on human health. Most of these edible flowers are commonly consumed, but some are less known due to limited distribution or seasonality. These lesser-explored flowers often contain compounds that offer significant health advantages. Therefore, this review focuses on exploring the characteristics, phytochemical composition, and bioactive compounds found in less commonly examined edible flowers. The flowers included in this review are peonies, forget-me-nots, frangipani, alpine roses, wild roses, hibiscus species, common lilacs, woodland geraniums, camellias, Aztec marigolds, kiri flowers, sunflowers, yucca flower, hollyhocks, and cornflowers. Due to their diverse biological activities, these flowers provide various health benefits and can be used to be incorporated into food and supplements or develop mainly cancer-fighting medications.

## 1. Introduction

Throughout history, flowers have been used for decoration and culinary purposes due to their unique characteristics. Floriphagia, the consumption of flowers and inflorescences, dates to ancient civilizations such as the Romans, who used violets, roses, and lavender in their sauce preparations. Native Americans regularly consumed pumpkin flowers, while in the Middle Ages, Europeans made beverages and sauces with dandelions and French prepared calendula sauces [1].

Edible flowers have long been used to prepare a variety of foods, thanks to their attractive colors and flavors that appeal to consumers. In addition, numerous studies have highlighted the nutritional and biological properties of different types of edible flowers, particularly their phytochemical compounds [2, 3]. These compounds possess biological benefits, such as antimicrobial, anti-inflammatory, antidiabetic, hepatoprotective, antihypertensive, nephroprotective, and antiulcerogenic effects [4], and include phenolic and lipidic substances. As a result, edible flowers have become an important ingredient in the food industry [5]. In fact, edible flowers containing phytochemicals of biological interest have the potential to be used in the design and development of functional foods or dietary supplements [6].

Worldwide, there are a huge number of edible flowers widely known. They are subjects of several studies, which include *Gardenia jasminoides*, *Viola × wittrockiana*, *Hibiscus sabdariffa*, *Rosa* spp., *Dahlia* spp., *Geranium* spp., *Lonicera* spp., *Nasturtium* spp., and *Magnolia* spp. [7].

There are a variety of edible flowers worth exploring, including peony (*Paeonia officinalis* L.), frangipani (*Plumeria alba*), true forget-me-not (*Myosotis scorpioides* L.), alpine rose (*Rosa pendulina*), wild rose (*Rosa canina*), some hibiscus species (*H. deflersii*, *H. micranthus*, and *H. calyphyllus*), common lilac (*Syringa vulgaris*), woodland geranium (*Geranium sylvaticum*), camellia (*Camellia japonica*), Aztec marigold (*Tagetes erecta*), kiri flower (*Paulownia fortunei*), sunflower (*Heliathus annuus*), yucca flower (*Yucca* spp.), hollyhock (*Alcea rosea*), or cornflower (*Centaurea cyanus*). These flowers possess important biological properties that have yet to be fully explored. Some have been used to extract and isolate bioactive compounds that have been tested to treat certain diseases. Therefore, this review aims to provide an analytical summary of the phytochemical composition and biological benefits of these edible flowers that have been less studied but can positively impact human health.

## 2. Are the Lesser-Explored Edible Flowers a Good Source of Bioactive Secondary Metabolites?

Lately, there has been a growing interest in edible flowers as a sustainable and functional food option. This is due to their potential as antioxidants and their ability to add color, aroma, and flavor to various dishes [3, 8]. Usually, edible flowers can be consumed in their entirety; however, in some cases, only their petals or buds are edible [9]. A wide range of flowers has been extensively studied, including popular ones like hibiscus that are consumed globally. In the past 5 years (2020–2024), 95 reviews discussing edible flowers have been published and indexed on the Web of Science database. These reviews largely focus on popular edible flowers, with 43% concentrating on Food Science and Technology and 32% examining their impact on human health. However, there is a limited selection of flowers typically used in local or regional cuisine or traditional medicine, and research is ongoing to uncover their potential applications.

**Figure 1:**
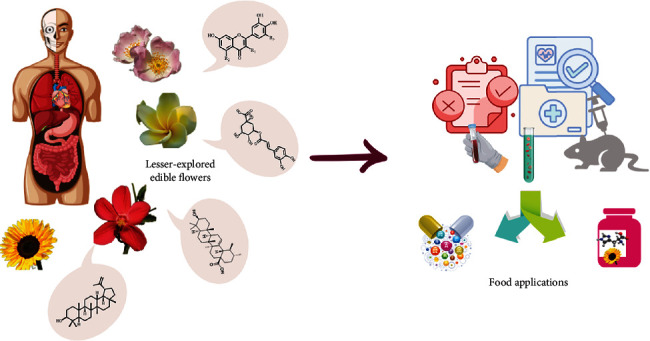
Representation of the duality of lesser-known edible flowers as sources of bioactive compounds.

Edible flowers are not only a source of essential nutrients, but they also contain bioactive compounds that can interact with living tissues, resulting in numerous positive effects [10]. In Figure 1, the duality of lesser-explored edible flowers is represented. These flowers are not only valuable from a nutritional standpoint but also serve as an important source of bioactive compounds. They can be used as raw materials to produce food supplements, as several scientific studies have demonstrated their effectiveness. Their chemical composition makes them highly beneficial for human health, and they can be used as adjuvants to treat various health conditions, such as joint inflammation, prevention of skin photodamage, and early suppression of malaria infections [11–13]. Studies have shown that consuming edible flowers can have a significant impact on overall health and well-being. Rescuing the importance of less-explored edible flowers is crucial for extending their use beyond local consumption. It also aids in the recovery of edible flower species that have been replaced and threatened by human activity [4].

On the other hand, the chemical composition of edible flowers includes a vast array of secondary metabolites that possess various biological activities. These metabolites are classified into families, including phenolic compounds, terpenes, terpenoids, steroidal substances, saponins, and cyanogenic glucosides.

Phenolic compounds encompass a vast range of secondary metabolites, which have reported diverse biological activities that depend on their chemical structure and concentration [14]. The polyphenol group is one of the largest classes of secondary metabolites, including flavonoids and phenolic acids as the main categories [15], frequently consumed in the diet due to their association with reduced inflammatory biomarkers and improved cardiovascular health. Polyphenol compounds act as donors of protons or electrons, resulting in a protective effect on cells damaged by free radicals, as a result, chronic illnesses are reduced [16]. Consuming polyphenols has been reported to regulate arterial pressure and improve serum biomarkers of endothelial function. Additionally, it decreases asymmetric serum dimethylarginine (AMDA) and oxidative low-density lipoprotein (LDL) [17]. Specifically, ADMA-related disorders can lead to fibrosis in the kidneys, heart, and liver [18].

The petals of lesser-explored edible flowers may contain different lipidic compounds, such as terpenes and terpenoids (including phytosterols), which have significant biological activities. Figure 1 shows some chemical structures of terpenes and terpenoids found in lesser-explored edible flowers, *β*-sitosterol one of the most common in them. These compounds have been found to possess a wide range of biological activities, including anticancer, antimicrobial, anti-inflammatory, antioxidant, and antiallergic properties [19]. Besides, triterpenoids have been extensively researched for their biological impacts, including anti-inflammatory, antimicrobial, antiviral, hepatoprotective, antidiabetic, and anticancer properties [20, 21]. They are contained in the essential oils of some edible flowers, such as frangipani, wild roses, woodland geranium, camellia, and Aztec marigold.

Several studies have reported a wide range of biological benefits linked with phytosterols, including reducing the levels of total and LDL cholesterol, which in turn results in decreased risk of cardiovascular diseases; antiobesity, antidiabetic, antimicrobial, anti-inflammatory, anticancer, and immunomodulatory properties [22]. Additionally, intake of phytosterols can help decrease lipid and cholesterol levels in the bloodstream and improve insulin resistance and lipid metabolism [20].

**Figure 2:**
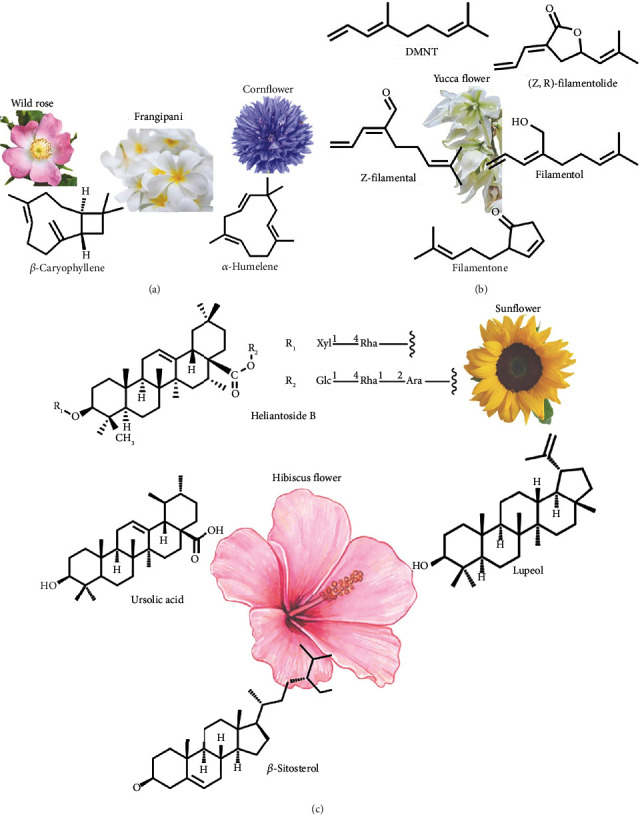
Chemical structures of the main terpenes and terpenoids found in some lesser-known edible flowers: (a) sesquiterpenes, (b) DMNT and tetranorsesquiterpenoid derivates, and (c) triterpenoids. An image is shown of the main edible flowers in which the compounds have been found.

Below is a discussion of valuable information on secondary metabolites found in edible flowers with various chemical compositions that have not been fully explored, but they are described to possess various biological activities.  Figure 2 shows images of these edible flowers, highlighting their unique features, such as their colors and morphologies.

### 2.1. Peony (*Paeonia officinalis* L.)

**Figure 3:**
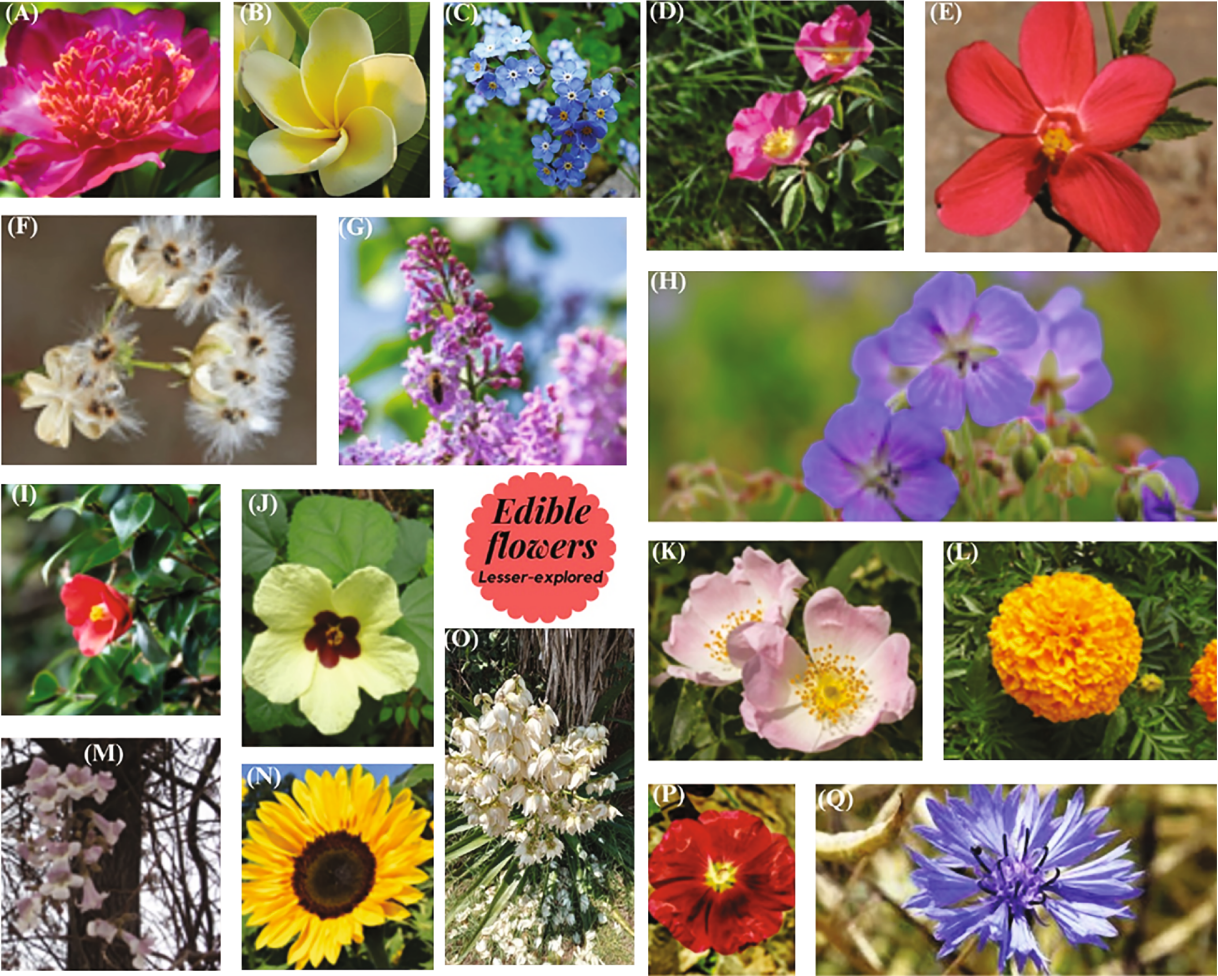
Images of lesser-explored edible flowers: (A) peony, (B) frangipani, (C) forget-me-not, (D) alpine rose, (E) crypt, (F) hibiscus, (G) common lilac, (H) woodland geranium, (I) camellia, (J) mallow, (K) wild rose, (L) Aztec marigold, (M) kiri, (N) sunflower, (O) yucca, (P) hollyhock, and (Q) cornflower.

It is a perennial plant (Figure 3(A)) that originates from Europe and is mostly grown in gardens [23]. Traditional medicines (Hindu and Chinese) have used peonies for their antihypertensive, neuroprotective, and antiulcerative properties. Additionally, certain red varieties of this flower are used in infusions to alleviate symptoms such as coughing, chest pain, and joint pain [13].

It is known for its high content of total phenolic compounds (Table 1). Recent studies have also shown that peony has high antioxidant activity, which is not solely attributed to its phenolic compounds, but may be due to other phytochemicals in the flower [24].

It is important to note that the bioactive compounds found in these flowers, which are of the greatest interest, are those that have antioxidant activity. Hence, emerging technologies have been employed to extract these compounds efficiently. One such technology is enzyme-assisted extraction, which is used to extract an ethanolic extract of peony petals. This resulted in a 3.6%–16% increase in the extraction of phenolic compounds (3985.5 mg GAE/100 g dry base) and an 11% rise in anthocyanins (720.5 mg cyanidin-3-O-glycoside/100 g dry base) compared to conventional extraction methods [13].

**Figure 4:**
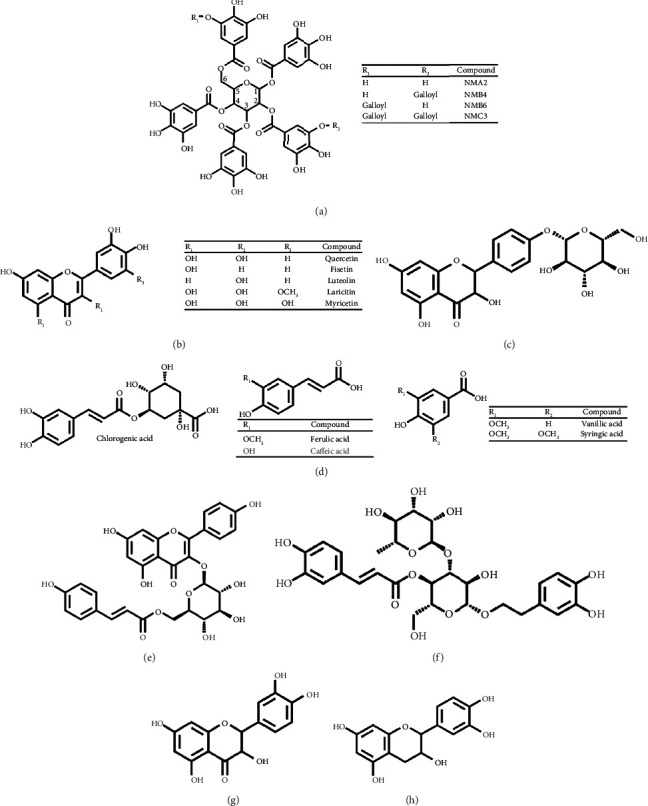
Chemical structures of the most important phenolic compounds found in lesser-known edible flowers: (a) galloyl glucosides; (b) flavonols; (c) dihydrokaempferol-4'-O-glucopyranoside; (d) phenolic acids; (e) tiliroside; (f) acteoside; (g) taxifolin; and (h) catechin.

Flower petals contain several galloylglucosides (Table 1) [39]. Their chemical structures are shown in Figure 4(a). NMA2 exhibits cytotoxic effects and inhibits cell proliferation in Mia-PaCa-2 pancreatic cancer cells by suppressing markers of pancreatic cancer stem cells, making it a potential treatment option [40].

Research has been conducted on the antimicrobial properties of ethanolic extracts from peony petals. It has been found that at a concentration of 6 mg/L, the extracts can inhibit the growth of *S. abony*, while for *E. coli*, *P. aeruginosa*, and *E. fecalis*, a concentration of 60 mg/L is needed [20], and concentrations between 2 and 256 mg/L for *K. pneumoniae* [40]. This is due to the secondary metabolites present in peony flowers. However, further research is necessary to determine the safety of peony flowers for human health.

### 2.2. Frangipani (*Plumeria alba*)

Frangipani flowers (Figure 3(B)) are found only in the West Indies and are highly regarded for their potential biological benefits (anti-inflammatory, analgesic, antimicrobial, antipyretic, and antioxidant properties), which have been evaluated in both fresh flowers and their essential oils. Traditionally, the flowers have been used in medicine to treat conditions such as fever, cough, diarrhea, and asthma [41].

Compounds that contain both essential oils and full flowers have been identified. The main compounds detected in the essential oil [25, 26] and full [27] of frangipani flowers are shown in Table 1. Syringic acid (Figure 4(d)) has been reported to have neuroprotective and hepatoprotective effects [42, 43]. Additionally, compounds with antibacterial properties have been found [42], which are displayed in Table 1.

Recently, Ferdosi et al. [28] reported that the methanolic extract of frangipani is effective in inhibiting the growth of several bacteria, including *E. coli* (up to 90%), *Salmonella* spp. (up to 84%), *Pseudomonas* spp. (up to 90%), *Bacillus* spp. (up to 93%), and *Staphylococcus* spp. (up to 92%). Additionally, the extract was found to inhibit the growth of five species of the fungus *Trichoderma*, including *T. viride* (90%), *T. harzianum* (86%), *T. hamatum* (77%), *T. reesei* (97%), and *T. koningii* (76%).

The essential oil extracted from frangipani flowers has been found to possess antibacterial properties against various types of bacteria, such as *S. aureus*, *E. fecalis*, *B. subtilis*, *S. pneumoniae*, *K. pneumoniae*, *P. aeruginosa*, and *E. coli* [41]; besides, it has been reported the potential to inhibit lipid peroxidation and biofilm formation of *P. aeruginosa* by up to 80% [41].

Moreover, the aqueous extracts of frangipani flowers have been utilized as a medium for the biosynthesis of silver nanoparticles [44] and chitosan–cadmium nanoparticles [39] that have demonstrated significant biological activities including antimicrobial, antioxidant, and insecticidal properties. All properties of these flowers show promise due to their bioactive compounds and potential for nanoparticle biosynthesis. However, further research is needed to address toxic compounds. *Plumeria alba* and *Myosotis scorpioides* L. are often confused due to their shared common name of forget-me-not, but they have distinct characteristics and properties.

### 2.3. True Forget-Me-Not (*Myosotis scorpioides* L.)

The true forget-me-not is a flower (Figure 3(C)) used in Nigerian traditional medicine to treat malaria infections, and scientists are studying its action mechanism to find scientific evidence of its effectiveness [11].

The ethanolic extract of forget-me-not was found to contain alkaloids, terpenes, tannins, flavonoids, saponins, and anthraquinones and has the ability to suppress early malaria infection (about 80% at 100 mg/kg of doses), 85.70% inhibition levels in the residual malaria infection and 66.73% in the curative tests [11]. These findings support the traditional use of forget-me-not extract as an antimalaria remedy.

### 2.4. Alpine (*Rosa pendulina*) and Wild Roses (*Rosa canina*)

These two types of roses (Figures 3(D) and 3(K)) have high levels of phenolic compounds (Table 1). The analysis of the phenolic compound profile shows that each type of rose has a different composition, with the alpine rose having higher levels of flavonoids, followed by benzoic acids and then flavones [24]. In contrast, the wild rose has more flavonoids, followed by cinnamic acids, flavones, and benzoic acids in lower quantities. Additionally, both flowers contain vitamin C (Table 1) [24].

The hydroalcoholic extract of wild rose contains eugenol, linalool, and rutin (quercetin-3-rutinoside, and its anxiolytic effect has been evaluated in the murin model, which acts on the GABA system and serotonin, thereby explaining this effect [45]. The essential oil of wild roses has been analyzed and identified by Öz and colleagues [29], and the composition profile is shown in Table 1, which includes aldehydes, terpenes, and terpenoids.

### 2.5. Hibiscus Species (*H. deflersii* Schweinf. ex Cufod, *H. micranthus* L., and *H. calyphyllus* Cav)

The hibiscus genus includes many species, with *H. sabdariffa* being the most extensively researched. However, other species, such as the crypt flower (*H. deflersii* Schweinf. ex Cufod), hibiscus (*H. micranthus* L.), and mallow (*H. calyphyllus*), have also gained attention in recent years for their unique morphologies and colors (Figures 3(E), 3(F), and 3(J)) and their beneficial properties.

Researchers found three important sterols in three varieties of edible flowers: ursolic acid, *β*-sitosterol, and lupeol (Figure 3(C)). The highest contents of these sterols were found in the petroleum ether fraction of the *H. deflersii* variety [46].

Studies have shown that ursolic acid (Figure 3(C)) has anticancer properties. It has been evaluated in vitro in cell lines HepG2 (hepatocellular carcinoma) and MCF-7 (breast cancer) by decreasing COX-2, inducing cell cycle G1/G2 arrest, and promoting apoptosis [46].

Among these fractions, the petroleum ether fraction of *H. deflersii* was particularly noteworthy for its strong activity, with IC_50_ values of 14.4 and 11.2 *μ*g/mL against the two cancer cells, respectively. This activity was even higher than the standard vinblastine, which had IC_50_ values of 3.48 and 5.44 *μ*g/mL against the same cancer cells [46].

*β*-Sitosterol (Figure 2(c)) has been found to exhibit anticancer properties against human colon cancer cells (HT116). The mechanism of action involves the induction of caspase 3/9 activation, along with an increase in cytochrome C release and a decrease in antiapoptotic protein Bcl-2 and mRNA expression [46].

Lupeol (Figure 2(c)) has been found to induce apoptosis and cell cycle arrest in the G0/G1 phase. This is achieved through downregulation of PI3-kinase, phosphoprotein kinase B, and cyclin D1 expression, as well as upregulation of p21 and p27 expression, upregulating the expression of miR-212-3p in osteosarcoma cells in vitro [46, 47]. Studies have shown that lupeol is effective against the MCF-7 and MG-63 cell lines (osteosarcoma) [47, 48]. This discovery suggests that it could be beneficial to use this treatment against cancer [46]. However, further studies are needed to understand the cytotoxic process fully.

### 2.6. Common Lilac (*Syringa vulgaris*)

The common lilac flowers are often cultivated throughout Europe as ornamental plants (see Figure 3(G)) and are utilized in the fragrance industry. They have been used in traditional medicine for their immunomodulatory properties and various biological activities, including antipyretic, anti-inflammatory, antioxidant, and antidiabetic effects. Traditionally, they have been used to treat gastrointestinal problems [30, 31]. The phenol and flavonoid contents vary (Table 1). Blue, rose, purple, and white flowers had the highest content of phenolic compounds, while the flavonoid content was lowest in white flowers [30].

The flowers were found to contain bioactive compounds such as phenylpropanoids [30, 31]; the profile is displayed in Table 1. Several polyphenols have been identified within quercetin-3-glucoside, quercetin-3-rutinoside, and acteoside, which have exhibited activity in preventing blood stasis syndrome, as evaluated in a rat model [49]. Blood stasis syndrome has been linked to gynecopathy and sensitivity to cold. Common lilac flowers exhibit important antioxidant activity. Ethanolic extracts demonstrated 66%–80% elimination of radicals through the DPPH assay, while the ABTS assay showed a radical scavenging activity ranging from 51.9%–72% [30].

A study was conducted using an ethanolic extract from lilac flowers to determine its cytotoxic activity on HeLa (human cancer cells) and B16F10 (murine melanoma cells) tumoral lines. The extract showed a decrease in the viability of cancer cells, but the effect was not dependent on the dosage. The results were similar to the positive control of cis-platinum. Interestingly, the anticancer activity was only observed in the flower extract and not in the extract of leaves or tree bark [30]. This suggests that the lilac flowers are the most valuable part of the plant as they have the potential as an alternative treatment for melanoma and prevent blood stasis syndrome.

### 2.7. Woodland Geranium (*Geranium sylvaticum*)

The woodland geranium (Figure 3(H)) is a plant that grows in the wild in some parts of Finland and Turkey. Its flower extracts have been used in traditional medicine to aid in relieving infectious diseases, such as antiviral or antimicrobial activity [32].

The woodland geranium flowers contain high levels of total phenols (Table 1), and the main is geranin, which has exhibited antioxidant, antitumor, and antiviral properties [50]. When the flower petals are fully open, the geranin content decreases by about 42% compared with the bottom phase [50]. Additionally, when the flowers are fully bloomed, quercetin and kaempferol diglucoside B have been detected at low levels.

The essential oil contains around 78 different components, including terpenoids such as linalool and limonene, aldehydes like benzaldehyde and 2E,4E-heptadienal, hydrocarbons including decane, dodecane, and tetradecane, and other compounds like 1-octen-3-ol, pentyl furan, and 1,2,4-trimethylbenzene [51]. Also, woodland geranium flowers were utilized to study the inhibition of acetylcholinesterase, which is commonly observed in drugs used to treat Parkinson's disease [52].

### 2.8. Camellia (*Camellia japonica*)

One of the most beautiful Asian flowers is camellia (Figure 3(I)), used in traditional oriental medicine. Its potential as an antioxidant and antimicrobial agent has been studied and found to be effective. Flowers and buds have been utilized to treat external and internal bleeding and reduce inflammation in various lesions [53].

Recently, Kong et al. [54] extracted camellia essential oil using hydro-distillation and identified its components, including alkanes, alcohols, carboxylic acids, and esters. The essential oil has inhibitory effects against microorganisms such as *S. aureus*, *E. coli*, *B. subtilis*, and *B. pumilus*. The antimicrobial properties of camellia essential oil can be attributed to the presence of long-chain alcohols, although it is possible that other components may also be influenced.

### 2.9. Aztec Marigold (*Tagetes erect*a)

The Aztec marigold is a flower (Figure 3(L)) that is known not only for its natural dyes but also for its potential health benefits. It has antioxidant properties with DPPH-IC_50_ (21.3–22.0 *μ*g/mL) and FRAP (78–112 *μ*mol Fe^2+^/g). Additionally, it has been shown to have a neuroprotective effect and the ability to delay aging [33].

Some reported therapeutic applications include treatments for muscle pain, liver health, and skin conditions such as wounds, burns, and eczema. It has also been used for issues such as poor eyesight, earaches, and hemorrhoids [55].

For instance, it has been found to have a high level of ascorbic acid (Table 1), which is more than what is found in some fruits like pear, watermelon, apple, and grape [56]. Ascorbic acid contributes to the Aztec marigold's antioxidant activity and aids in the prevention of anemia, enhances the absorption of iron, promotes collagen synthesis, and has an antitumoral effect through apoptosis. Additionally, it helps to control infections and can be used as a treatment for neurodegenerative illnesses [57].

Studies have found that Aztec marigold flowers' hydroalcoholic extract and its essential oil contain the antioxidant carotenoid lutein, which is the main responsible for some biological activities [58]. Lutein (Figure 4(b)) and its glycosides present in this flower offer protection against oxidative stress.

Additionally, myricetin (Figure 4(b)) and other flavonoids such as quercetin (Figure 4(b)), quercetagetin, and 6-hydroxykaempferol have been identified, which help reduce oxidative damage [33, 58] and have been shown to be effective against liver cancer (HepG2) and lung cancer (A549) cell lines [58].

Meurer et al. [59] administered lutein extracted from Aztec marigolds (8.2% lutein) to male Swiss mice with ulcerative colitis, and the dose of 300 mg/kg was the most effective in reducing the activity index of the disease, which was induced by adding dextran sulfate. In this in vivo study, it was observed that there was a decrease in the migration of inflammatory cells, resulting in reduced damage from ulcerative colitis. This positive effect was due to the reduction of inflammation processes.

Furthermore, Kazibwe et al. [34] have explored the potential of Aztec marigold aqueous extracts obtained through ultrasound-assisted extraction, and its composition is shown in Table 1. as well as exhibiting antioxidant activity (140 *μ*mol trolox equivalent/g) and antimicrobial activity against *S. mutans* and *P. aeruginosa* (less than 2x10^2^ CFU/mL for both).

Moliner et al. [33] have identified phenolic compounds in ethanolic extract from Aztec marigolds, specifically in orange and yellow varieties of flowers. Digallic acid, myricetin-di-hexoside, and laricitin (Figure 3(B)) and their derivatives (hexoside, di-hexoside, and galloyl hexoside) were tentatively identified and quantified within this group of compounds.

Also, the effects of flower ethanolic extracts on the viability of *C. elegans* were evaluated. The results showed that the extracts had no negative impact on worm viability compared to the control group. In fact, at a concentration of 2000 *μ*g/mL, the viability of the worms exceeded 90%, which was higher than the control group and demonstrated potential as a neuroprotective agent [33].

Additionally, these flowers exhibit antifungal (shown to be effective against *C. albicans*, *A. niger*, *A. flavus*, and *P. chrysogenum*) and anticancer activity in the MCF-7 cell line [58].

### 2.10. Kiri Flower (*Paulownia fortunei*)

Researchers have studied the effects of the ethanolic extract of the kiri flower (Figure 3(M)), on mice with a diet high in lipids and with conditions such as steatosis, obesity, and insulin resistance [35].

The kiri flower's ethanolic extract is rich in flavonoids [35], particularly apigenin and luteolin-7-O-glycoside (Table 1). The in vivo assays conducted on mice showed that the extract improved hepatic lipid accumulation, resulting in a decrease in triglycerides by 38.2%–241.79%, an increase in high-density lipoprotein (HDL) by 9.5%–12.5%, and a decrease in LDL by 37%–41%. The extract also helped decrease insulin resistance levels by 23.11%–29.91% in the blood. Moreover, the extract reduced the levels of phosphorylated AMPK, which plays a role in lipidic metabolism, preventing overaccumulation of these compounds. In this study by Lui and colleagues [35], mice treated with the extract decreased body weight up to 20%, indicating that the extract has a protective effect against obesity, which could aid in treating or preventing illnesses related to lipidic alterations.

In a study by Wang et al. [60], a PFFPS (*Paulownia fortunei* flower polysaccharide) was extracted from kiri flowers. The researchers tested PFFPS on chickens and discovered that even at lower doses, it could significantly boost the development of immune organs, increase leukocyte quantity and lymphocyte ratio, enhance antibody titers against Newcastle disease virus (NDV), and increase concentrations of IL-2 and IFN-*γ*, as well as the content of SIgA in the duodenum. Additionally, PFFPS was found to relieve immunosuppression brought about by cyclophosphamide (CTX). These findings indicate that PFFPS has the potential to be a valuable component for new immunopotentiators and/or adjuvants in the livestock and poultry industries.

### 2.11. Sunflower (*Heliathus annuus*)

The sunflower (Figure 3(N)) is renowned for its unique heliotropism and has been a culinary ingredient for over 3000 years. The petals of the flower are a popular addition to salads [61] and can be eaten raw when they are in the young bud stage. In this case, they have an artichoke flavor but can also be cooked [62]. The flower has been linked to biological benefits, and it has been reported to have a high total phenolic compound content (Table 1) [63].

The flower petals of sunflowers contain triterpene glucosides, which include helianthoside. Researchers evaluated the anti-inflammatory activity of these compounds and found that heliantoside B (Figure 2(c)) had the highest activity, with an inhibitory ratio of 80% [36].

Additionally, researchers have tested the effects of an ethanolic extract from sunflowers on skin damage caused by ultraviolet radiation in normal human fibroblasts, which were exposed to UV radiation to induce photodamage. After exposure, the cells were treated with the ethanolic extract at 100 *μ*g/mL concentration. The study is aimed at evaluating the extract's ability to protect against photoaging, inflammation, and photocarcinogenesis [62].

The ethanolic extract was found to significantly decrease the production of reactive oxygen species and the secretion of IL-6 induced by ultraviolet radiation by 56.96%. Additionally, it was observed that the extract reduced the secretion of vascular endothelial growth factor (VEGF) by 80.47%. After radiation exposure, concentrations of cyclooxygenase-2, nitric oxide synthase, and tumor necrosis factor-alpha (TNF-*α*) were notably increased in cells. However, treatment with doses of 100 *μ*g/mL of the extract reduced these concentrations by 26.99%, 29.34%, and 30.70%, respectively, compared to the control group [12]. These findings suggest that the ethanolic extract from sunflower may be an effective treatment for skin photodamage and related skin illnesses.

### 2.12. Yucca Flower (*Yucca* spp.)

The yucca flowers (Figure 3(O)) are consumed by people as seasonal food in arid and semiarid regions. There are different species within the *Yucca* genus. The flowers of *Y. filifera* have been found to contain antinutritional compounds, such as trypsin inhibitors (3.77 trypsin inhibited units/mg of flower), and hemolytic activity at Level 4, which is higher compared to other edible flowers [64].

One example of a substance that has been researched for its potential to reduce proinflammatory substances, such as cytokines, is saponins [65]. However, saponins' effects in yucca flowers have not yet been studied. Meanwhile, *Y. elephantipes* petals contain various phenolic compounds (Table 1). These phenolic compounds are valuable for their various biological properties, such as acting as antioxidants [15], regulating arterial pressure [16], and potentially preventing cancer [66].

Recently, Tröger et al. [37] conducted a study on the volatile compounds found in yucca flowers. They analyzed six different species of yucca and identified acetogenin hydrocarbons and terpenoids as the main chemical components. Specifically, they found acetogenin hydrocarbons (Table 1) and tetranorsesquiterpenoids (E-filamentol, filamentolide, filamental, and filamentone as the most important terpenoids. These compounds are novel natural products found in yucca flowers and are considered oxygenated derivatives of (e)-4,8-dimethyl-1,3,7-nonatriene (DMNT).  Figure 2(b) displays the chemical structures of these tetranorsesquiterpenoids.

### 2.13. Hollyhock (*Alcea rosea*)

Hollyhock is a popular ornamental flower in Egyptian gardens (Figure 3(P)). Its use in medicine for treating hyperglycemia and hypolipidemia is a long-standing tradition. It has been known to possess diuretic, immunomodulatory, analgesic, anti-inflammatory, cooling, demulcent, emollient, febrifuge, astringent, and antiulcer properties since ancient times [67, 68].

Among the flavonoids found, dihydrokaempferol and kaempferol-3-O-*β*-d-glucopyranoside have been found to have significant antioxidant activity, while apigenin stimulates the production of compounds related to the immune system [69]. Hollyhock flowers also contain a compound called dihydrokaempferol-4⁣′-O-glucopyranoside (Figure 4(c)), which has potent antioxidant and anticancer properties that have been tested on HepG-2 cancer cells [70].

### 2.14. Cornflower (*Centaurea cyanus*)

Cornflower has garnered attention due to its natural blue color (Figure 3(Q)) and is primarily used for dish decoration. However, it is also a significant source of bioactive compounds that have been less explored [71].

Researchers have examined the biological compounds present in cornflowers, and they found that succinic acid has the highest concentration among the organic acids (2.55 g/100 g) and may play a role in energy production. Additionally, the total tocopherol content was reported at 1.30 mg/100 g on a dry basis [72], 6.73% of the recommended daily intake for this vitamin.

Lockowandt et al. [38] identified 12 flavonoids and 4 anthocyanins as part of their study on phenolic compounds. The flower's ethanolic extracts exhibited potential antibacterial activity against Gram-positive bacteria, including *L. monocytogenes* and methicillin-susceptible strains.

A recent study on cornflowers found over 30 volatile compounds in their petals, with sesquiterpenes being the most prevalent group of compounds [73]; and the dominant ones are related to the therapeutic effect of these flowers, the contents are shown in Table 1, and their chemical structures are displayed in Figure 2(a).

## 3. Potential Uses for Lesser-Explored Edible Flowers in Food Science and Technology

As described previously, incorporating edible flowers into one's diet or using them as an ingredient in food or products can offer various health benefits to consumers [74]. This makes them a viable alternative for raw materials in producing supplements, nutraceuticals, and beverages. Additionally, these compounds can be extracted and used as the basis for food supplements or even drugs.

Edible flowers are known for their vibrant colors, which serve to attract pollinators. These colors come from natural pigments like chlorophylls, carotenoids, and anthocyanins. This visual aspect is advantageous in using edible flowers as raw material, as the color contributes to the appeal and quality of the final food product [57].

In this sense, the hydrophilic extracts from three different edible flowers, namely rose, cornflower, and dahlia, have been explored as possible replacements for anthocyanin extract in yogurts. These extracts have exhibited comparable qualities to a control yogurt, making them a viable alternative for use [75]. Red powders obtained from edible flowers, such as cornflowers, have been microencapsulated and developed as additives for food products like sweets, jellies, puddings, drinks, and dietary supplements [76]. The purpose of these microencapsulated foods is to maintain their antioxidant properties.

Innovative products have been created using powders from edible flowers. Mortas [68] suggested a milk-based powder from hollyhock flower extract created through spray drying. This ready-to-drink product is derived from a medicinal plant and aims to promote food sustainability.

Terpenes and terpenoids are several important compounds found in the essential oils of edible flowers, which are discussed in this review. These compounds are responsible for the essential oils of numerous plants and have been found to exhibit antimicrobial activity. As a result, they can be used as preservatives in the food industry.

In the context of food applications, essential oils from *Paenoia suffruticosa* flower buds have been found to have effective antimicrobial properties against common foodborne pathogens. This suggests they could be used as a natural antimicrobial agent in foods [77]. Additionally, recent reports have shown that frangipani extracts exhibit antibacterial activity against microorganisms such as *E. coli*, *P. aeruginosa*, *S. aureus*, *C. albicans*, *S. pyogenes*, *S. typhi*, *B. pumilus*, *B. subtilis*, *S. dysenteriae*, *P. mirabilis*, *P. fluorescens*, *B. cereus*, *S. flexneri*, and *M. furfur*. Furthermore, these extracts contain a significant amount of antioxidant compounds such as phenols and flavonoids, which suggests their potential use as antibacterial agents for pharmaceutical purposes [78].

On the other hand, *Myosotis scorpioides* L. has been traditionally used in medicine. It is infused to treat various conditions, such as bronchitis, malaria, and inflammation, and has shown microorganism-suppressing effects [11].

Some wild rose species, such as *Rosa micrantha*, are valuable for food additives because they contain high levels of tocopherols and ascorbic acid, which are even higher than those found in citrus fruits. This makes their antioxidant activity important for additives. In addition, the carotenoid pigments found in these roses could be used as a natural and profitable source of food coloring [79].

Several edible flower species such as the tiny flower hibiscus (*Hibiscus micranthus* L.), common lilac (*Syringa vulgaris* L.), pink geranium (*Pelargonium graveolens*), tea flower (*Camellia sinenses* L.), and kiri flower (*Paulownia fourtunei*) have shown potential antimicrobial and beneficial properties for food applications. The tiny flower hibiscus demonstrated significant inhibition of *S. pyogenes*, *S. aureus*, and *S. pneumonia* [80]. Infusions of common lilac flowers contain bioactive compounds such as phenols and organic acids, making them valuable for enriching the diet of consumers in a natural way [30].

Pink geranium extracts have demonstrated antimicrobial activity, while essential oils have shown efficacy against *E. coli* and significant antiviral activity. This suggests that these flowers could be used in the food industry as a natural preservative. Importantly, these substances are considered nontoxic as they did not produce mutagenicity effects in tests, making them harmless [81].

Additionally, the tea flowers are not very studied, however, special attention has been paid to them, because they are used to make tea, and it has been highlighted that these preparations are rich in carbohydrates and some phenolic compounds, and in animal models it has regulated intestinal homeostasis and stimulated immunoregulation, which is why it is considered a food with functional potential, implementing it regularly in the diet [82].

Furthermore, not much research has been conducted on tea flowers. However, they have garnered special attention due to their use in tea making. Studies have revealed that these preparations are rich in carbohydrates and certain phenolic compounds. In animal models, it has been shown to regulate intestinal balance and stimulate immune regulation. Because of these findings, tea flowers are considered to have functional potential as a food item, and it is recommended to include them regularly in the diet.

The essential oil of kiri flowers has been analyzed and found to contain 31 volatile components known for their antibacterial and antioxidant properties. Additionally, it has been reported to have significant inhibitory potential against xanthine oxidase, making it advantageous for the development of antioxidant compounds targeted at proteins in the food industry [83].

It is important to note that certain edible flowers and inflorescences can harm humans. Therefore, it is crucial to identify the nutritional properties of flowers and inflorescences and determine the presence of antinutrients, toxins, and allergens to ensure the safety of consuming nonconventional flowers in the human diet because the specific compounds responsible for beneficial effects remain unknown. Many of these compounds possess multiple bioactivities and may have promising applications in the healthcare industry.

## 4. Conclusions

Many types of edible flowers have not been thoroughly studied. However, they contain a significant number of bioactive metabolites that may offer various health benefits. Most of them have been used as food in some cultures, but their chemical study and biological activities have been minimally explored. The lesser-known flowers could be considered good sources of compounds that have benefits for human health. By examining these flowers' properties and metabolite content, they can be recommended for use in the food industry, as supplements, nutraceuticals, or in the development of functional foods. Their use can also be recommended in the development of anticancer medications.

## Figures and Tables

**Table 1:** Main phytochemicals found in lesser-explored edible flowers.

**Edible flower**	**Main phytochemicals**	**Ref.**
Peony	Flowers: TPC^a^ (1930.5), derivatives of PGG or NMA2: NMB4, NMB6, 3,6-bis-O-digalloyl-1,2,4-tri-O-galloyl-*β*-D-glucose (NMC3)	[23, 24]

Frangipani	Essential oil^b^: benzyl salicylate (34.0), benzyl benzoate (12.4), germacrene B (10.30), limonene (9.1), linalool (7.9), *α*-cedrene (8.0), caryophyllene oxide (7.9), and (E,E)-*α*-farnesene (6.6). Flowers (db)^b^: 2,3-dihydrobenzofuran or coumaran (27.95), 9-octadecyne (14.29), cyclononanone (14.59).	[25, 26]
	Minor components^c^: quercetin and luteolin (96.9), cyanidin-3-glucoside (5208.9), syringic acid (6501.8).	[27]
	Compounds with antibacterial activity^b^: 2-hydroxycyclopent-2-en-1-one (0.87), phenol (1.04), benzyl alcohol (5.57), catechol (0.99), and hydroquinone (1.01).	[28]

Alpine rose	TPC^a^ (1773.7), vitamin C^d^ (7.2)	[24]

Wild rose	Flowers (db): TPC^a^ (1396.6), vitamin C^d^ (12.2).	[24]
	Essential oil^b^: transgeraniol (42.1), linalool (11.2), caryophyllene (6.07), tricosane (5.35), nonanal (3.33), and (E)-2-hexenal (3.81)	[29]

Common lilac	Flowers (db): TPC^d^ (1840-6710), flavonoids^d^ (210-270), syringin^c^ (14.64), acteoside^c^ (9408.78), echinacoside^c^ (7417.81), catechin^c^ (620.1–3019.0), chlorogenic acid^c^ (492.4–7825.9), ferulic acid^c^ (6.5-944.2), quercetin^c^ (7642.07) and kaempferol-glucoside^c^ (3814.93). Secoiridoids^c^: secologanoside (27.66), oleuropein (6744.01) and dimethyl oleuropein (35729.89).	[30, 31]

Woodland geranium	Flowers (db): TPC^a^ (1267.8)	[32]

Aztec marigold	Flowers (db): ascorbic acid^d^ (25.46-36.69)	[33]
	Aqueous extract: TPC^a^ (220) and flavonoids^e^ (300)	[34]

Kiri	Ethanolic extract^b^: apigenin and luteolin-7-O-glycoside (67.78)	[35]

Sunflower	Flowers (dry basis): TPC^a^ (1685)	[36]

Yucca *Y. elephantipes*	Flowers (db)^c^: rutin (393.2), vanillic acid (205.28), ferulic acid (140.5), caffeic acid (74.1), chlorogenic acid (47.52), kaempferol (37.16), protocatechuic acid (21.14), quercetin (9.54), and gallic acid (5.28).	[2]
	(Z)-7-hexadecene (7Z-C16ene), (6Z,9Z)-6,9-heptadecadiene (6Z,9Z-C17diene), (Z)-8-heptadecene (8Z-C17ene), (Z)-9-octadecene (9Z-C18ene), and (Z)-9-nonadecene (9Z-C19ene), (E)-filamentol (4-hydroxymethyl-8-methyl-1,3,7-nonatriene), filamentolide, filamental (2-allylidene-6-methylhept-5-en-1-al), and filamentone	[37]

Cornflower	Volatile compounds in flowers^b^: *β*-caryophyllene (26.17) and *α*-humelene (9.77).	[38]

Abbreviations: db, dry basis; GAE, gallic acid equivalents; NMB4, 3-O-digalloyl-1,2,4,6-tetra-O-galloyl-*β*-D-glucose; NMB6, 6-O-digalloyl-1,2,3,4-tetra-O-galloyl-*β*-D-glucose; NMC6, 3,6-bis-O-digalloyl-1,2,4-tri-O-galloyl-*β*-D-glucose; PGG or NMA2, 2,3,4,6-penta-O-galloyl-*β*-D-glucose; TPC, total phenolic compounds.

^a^mg GAE/100 g.

^b^percentage (%).

^c^ *μ*g/g.

^d^mg/100 g.

^e^mg rutin/100 g.

## Data Availability

Data sharing is not applicable. The article describes entirely theoretical research.
